# A Combination of Extreme Environmental Conditions Favor the Prevalence of Endospore-Forming Firmicutes

**DOI:** 10.3389/fmicb.2016.01707

**Published:** 2016-11-03

**Authors:** Sevasti Filippidou, Tina Wunderlin, Thomas Junier, Nicole Jeanneret, Cristina Dorador, Veronica Molina, David R. Johnson, Pilar Junier

**Affiliations:** ^1^Laboratory of Microbiology, University of NeuchâtelNeuchâtel, Switzerland; ^2^Vital-IT group, Swiss Institute of BioinformaticsLausanne, Switzerland; ^3^Laboratorio de Complejidad Microbiana y Ecología Funcional and Departamento de Biotecnología, Facultad de Ciencias del Mar y Recursos Biológicos, Universidad de AntofagastaAntofagasta, Chile; ^4^Centre for Biotechnology and Bioengineering, CeBiB, University of ChileSantiago, Chile; ^5^Departamento de Biología, Facultad de Ciencias Naturales y Exactas. Universidad de Playa AnchaValparaíso, Chile; ^6^Department of Environmental Microbiology, Swiss Federal Institute of Aquatic Science and Technology (Eawag)Dübendorf, Switzerland

**Keywords:** endospores, Firmicutes, qPCR, spo0A, 16S rRNA gene, *Clostridium*, geothermal springs, mineral springs

## Abstract

Environmental conditions unsuitable for microbial growth are the rule rather than the exception in most habitats. In response to this, microorganisms have developed various strategies to withstand environmental conditions that limit active growth. Endospore-forming Firmicutes (EFF) deploy a myriad of survival strategies in order to resist adverse conditions. Like many bacterial groups, they can form biofilms and detect nutrient scarcity through chemotaxis. Moreover, within this paraphyletic group of Firmicutes, ecophysiological optima are diverse. Nonetheless, a response to adversity that delimits this group is the formation of wet-heat resistant spores. These strategies are energetically demanding and therefore might affect the biological success of EFF. Therefore, we hypothesize that abundance and diversity of EFF should be maximized in those environments in which the benefits of these survival strategies offsets the energetic cost. In order to address this hypothesis, geothermal and mineral springs and drillings were selected because in these environments of steep physicochemical gradients, diversified survival strategies may become a successful strategy.We collected 71 samples from geothermal and mineral environments characterized by none (null), single or multiple limiting environmental factors (temperature, pH, UV radiation, and specific mineral composition). To measure success, we quantified EFF gene copy numbers (GCN; *spo0A* gene) in relation to total bacterial GCN (16S rRNA gene), as well as the contribution of EFF to community composition. The quantification showed that relative GCN for EFF reached up to 20% at sites characterized by multiple limiting environmental factors, whereas it corresponded to less than 1% at sites with one or no limiting environmental factor. Pyrosequencing of the 16S rRNA gene supports a higher contribution of EFF at sites with multiple limiting factors. Community composition suggested a combination of phylotypes for which active growth could be expected, and phylotypes that are most likely in the state of endospores, in all the sites. In summary, our results suggest that diversified survival strategies, including sporulation and metabolic adaptations, explain the biological success of EFF in geothermal and natural springs, and that multiple extreme environmental factors favor the prevalence of EFF.

## Introduction

Representatives of the Phylum Firmicutes have been known since the dawn of microbiology. Robert Koch when studying Anthrax, described the first Firmicute species: *Bacillus anthracis* (Blevins and Bronze, 2010). Since then, this microbial group has not ceased to amaze microbiologists because of the large functional diversity and the sophisticated survival strategies displayed by its members. Probably the best studied of these survival strategies is the formation of a *spore*, a structure that contains and protects the genetic material of the bacterium. Endospores have been claimed to be the most resistant cellular structures on Earth (Abecasis et al., 2013). As endospores remain viable for long periods of time, EFF can bear long-distance transportation, leading to a higher dispersal rate than other bacteria (Roberts and Cohan, 1995; Martiny et al., 2006). Although endosporulation is considered to be a critical survival mechanism (Stephens, 1998), EFF can also deploy other survival strategies. Those include motility, chemotaxis, DNA uptake, transformation, or biofilm formation (Errington, 1993). In addition, EFF display a large functional diversity and are, therefore, involved in a variety of ecosystem functions (Mandic-Mulec and Prosser, 2011). A multitude of metabolisms can be found among Firmicutes, including aerobic and anaerobic respiration (e.g., sulfate-reduction), acetogenesis, fermentation or phototrophy (Béjà et al., 2002; Logan, 2012; Galperin, 2013). Likewise, different species of Firmicutes display diverse ecological optima.

Improved persistence due to the combination of sporulation and other survival mechanisms, together with metabolic diversity, might offer EFF an ecological advantage among bacteria. This is supported by studies leading to the isolation of EFF in a multitude of environments (Martiny et al., 2006). For many of these habitats EFF might be found in the state of endospores (Nicholson et al., 2002; Fajardo-Cavazos and Nicholson, 2006; Zeigler, 2013). This is illustrated by, for example, the isolation of thermophilic species in artic sediments (Hubert et al., 2009, 2010) or in cool soil (Marchant et al., 2002, 2008). However, in other cases EFF would be found in a physiologically active state as vegetative cells. This is the case, for example, of thermophilic EFF species previously isolated and described from diverse geothermal environments (Derekova et al., 2007; Atanassova et al., 2008). In contrast to these examples based on culturing and isolation, molecular ecology studies have, paradoxically, failed to detect EFF. Comparative environmental genomic data has revealed the under detection of this group (von Mering et al., 2007). There are at least two potential explanations to this. On the one hand, a methodological bias against molecular detection of Firmicutes could explain their under detection (Filippidou et al., 2015) and, accordingly, it has been shown that a tailored DNA extraction method allows for a better assessment of the abundance and diversity of EFF in environmental samples (Wunderlin et al., 2013). On the other hand, the poor detection in molecular ecology studies might accurately reflect the relative distribution of EFF in environmental samples. In case of the latter, it is possible that the energetic demands of their survival strategies are a burden that limits the prevalence of EFF under non-limiting conditions. Indeed, bacteria that withstand harsh environmental conditions, have extra genes or even extra chromosomes (Barton, 2005), which results in a decrease in fitness under mesophilic conditions (Pope et al., 2010). Larger chromosomes resulting in decrease in fitness are also observed in endospore-forming Firmicutes: when there is no environmental pressure for sporulation, Firmicutes tend to lose their extra, sporulation-related genes (Callahan et al., 2008; de Hoon et al., 2010). Besides the energetic burden involved in the replication and maintenance of a larger genome, sporulation itself is a costly procedure, chosen only as a last resort adaptation (Ratcliff et al., 2013; Siebring et al., 2014). This would suggest that the biological cost of increased survival might be a better explanation of the distribution patterns of EFF in the environment. However, this possibility has not been previously addressed. In this study, we tested this hypothesis by measuring the relative abundance and diversity of EFF compared to the abundance of total bacteria in a series of mineral springs characterized by null, single or multiple limiting environmental factors. Abundance was estimated by measuring the gene count numbers (GCN) for either a spore-specific gene marker (*spo0A* gene), or a general bacterial gene marker (16S rRNA gene). The selection of *spo0A* as molecular marker for sporulation is a compromise as this gene has been in some cases found outside EFF (Galperin et al., 2012), but has been identified as the most prevalent gene among the genomes of known EFF (Bueche et al., 2013). For the first time, we present experimental evidence for the ubiquity of this group and we propose an ecological explanation for the under-detection of EFF in microbial community studies.

## Materials and Methods

### Sampling and Environmental Factors

To study the effect of limiting environmental factors on the presence of EFF, four environmental factors were investigated in this study: temperature, pH, UV radiation, and mineral composition of the springs. These factors were selected *a priori* before sampling. Samples were collected from 24 sites in Chile, Colombia, Germany, Greece, France, and Switzerland from 2011 to 2013 (**Supplementary Figure 1**). Temperature and pH were measured on site. Exposure to UV radiation was assessed by the exposure and the non-exposure of each site to sunlight. For the mineral composition of each spring, previously published data was used (Fytikas et al., 1986; Teschner et al., 2007). The categorization of the measures into limiting or non-limiting was based on reference physicochemical parameters and ranges considered to describe mesophilic conditions (**Supplementary Table 1**). This included temperature (20 to 55°C), pH (5.5 to 8.5), atmospheric pressure (∼1 atm), exposure to UV radiation and concentration of cell-toxic chemical compounds.

### DNA Extraction

Water samples were filtered through 0.22 μm membranes to collect biomass. Soil, sediment, and biofilm samples were subjected to an indirect DNA extraction procedure as previously described (Wunderlin et al., 2013). This modified protocol included the separation of the biomass from soil and sediment using a 1% (w/v) hexa-meta-phosphate solution followed by the collection of the biomass for DNA extraction. DNA was extracted using the FastDNA Spin Kit for Soil (MP Biomedicals, California). In order to ensure that DNA is not only extracted from vegetative cells but also from spores and other cells difficult to lyse, the first step of the extraction protocol was modified to include three sequential bead-beating steps. After each step, the sample was treated independently, allowing for DNA extraction of easy-to-lyse cells (first bead-beating round and downstream processing), harder cell wall cells, or other structures (second bead-beating), and resistant structures such as spores (second and third bead-beating). This method ensures that DNA from all cell types is extracted, while minimizing degradation of the DNA released in the previously bead beating round, which would compromise the representation of certain bacterial groups in the analysis. The final extracts were pooled together by ethanol precipitation (Wunderlin et al., 2013). Final DNA concentration was measured with a Qubit Fluorometer using a dsDNA HS Assay Kit (Invitrogen, California). The concentration of all samples was adjusted by dilution in sterile water to a concentration of 2 ng/μl.

### Quantitative PCR Assays

Prior to quantification, the integrity of 16S rRNA and *spo0A* (transcriptional factor responsible for the initiation of sporulation) genes was verified by PCR amplification of the complete genes (approximately 1500 bp for the 16S rRNA gene and 600 bp for *spo0A*). PCR amplification of the 16S rRNA gene was performed using the primer set GM3F and GM4R, as published previously (Muyzer et al., 1995), while for *spo0A* amplification a set of specific primers (spo0A166f and spo0A748r) was used as described previously (Wunderlin et al., 2013). In order to estimate the relative abundance of EFF in the bacterial communities of each sample, two quantitative PCR (qPCR) assays were used. First, for quantifying gene copy numbers (GCN) of total bacteria, qPCR amplification of the V3 hyper-variable region from the 16S rRNA gene was carried out. The primers used were 338f (5′-ACTCCTACGGGAGGCAGCAG-3′) and 520r (5′-ATTACCGCGGCTGCTGG-3′) (Muyzer et al., 1995; Bakke et al., 2011) and amplification was carried out under conditions previously described (Bueche et al., 2013). For the quantification of GCN for EFF, a primer pair targeting the *spo0A* gene was used as previously described (Bueche et al., 2013). Reactions were carried out in a final volume of 10 with 5 μl Rotor-Gene SYBR green PCR master mix (Qiagen, Germany), 1 and 0.45 μM of primers spo0A655f and spo0A834r, respectively, and 4 ng of DNA template. Amplification conditions were previously described (Bueche et al., 2013). All reactions were performed in triplicates. The standard curves for quantification of both V3 16S rRNA gene and *spo0A* gene were prepared from 10-fold dilutions (10^8^ to 10^2^ copies/μl) of a plasmid in which the V3 16S rRNA and *spo0A* gene of *B. subtilis* was inserted, respectively. The TOPO TA cloning kit (Invitrogen, California) was used to produce this plasmid in One Shot TOP10F’ chemically competent *E. coli* cells (Invitrogen, California), following the manufacturer’s guidelines. Plasmid DNA was extracted with the Wizard Plus SV Miniprep DNA purification system (Promega, Wisconsin) following the manufacturer’s instructions. DNA quantification was carried out with a Qubit 2.0 fluorometer and assay kit (Invitrogen, California) and the number of gene copies was calculated based on this quantification. The relative abundance of EFF in the samples was calculated as the ratio of the GCN values obtained from the qPCR assays (*spo0A*/16S rRNA gene copies). For both qPCR assays, quantification of the genes was performed using the ΔCt method.

### Amplicon Pyrosequencing and Analysis

In order to study the diversity of the bacterial communities and more specifically the diversity of EFF, eight samples were selected for 454 pyrosequencing using the services of Eurofins MWG Operon (Germany). Libraries were generated for both the 16S rRNA and the *spo0A* genes. The reason for sequencing both markers is that while the 16S rRNA gene provides phylogenetic information on the prevalence of Firmicutes relative to other bacterial groups, sporulation is a trait that is not shared by all Firmicute representatives (Onyenwoke et al., 2004). Therefore, in order to analyze the diversity of EFF, a functional marker related to sporulation, such as the *spo0A* gene is required. For the 16S rRNA gene, fragments of approximately 500 bp were retrieved using primers Eub8f (5′-AGAGTTTGATCCTGGCTCAG-3′) and Eub519r (5′-GTATTACCGCGGCTGCTGG-3′), as previously described (Li et al., 2009). Raw sequence data was analyzed with QIIME (Caporaso et al., 2010), using the pipeline for *de novo* OTU picking and diversity analyzes from 454 data suggested in QIIME tutorials. Amplicon sequencing resulted in a total of 117,542 sequence reads after quality filtering. Sequences were de-noised with the split_library.py function implemented in QIIME, and checked for chimera using USEARCH version 6.1 with the reference database used in the version 1.8.0 of QIIME. 2365 chimeric sequences were detected and removed from further analysis. To the rest of the trimmed and processed sequences, alignment was performed through the RDP^1^ using Infernal Aligner (Nawrocki and Eddy, 2007). OTUs were identified using a threshold of 97% sequence similarity with USEARCH version 6.1. Alpha diversity within the samples was calculated in rarefied subsets sequences to have equal sequence coverage (7302 sequences per sample) following the tutorial suggested by QIIME for 454-sequencing analysis. The parameters retained for the analysis were Richness, Shannon and Simpson diversity indices, and the percentage of the ratio OTUs/chao1 (coverage). The same analyses were performed after selecting solely the sequences assigned to the phylum Firmicutes. In this case, alpha diversity was calculated based on 1,188 sequences (rarefied samples).

For *spo0A* amplicon pyrosequencing a 602 bp sequence of the *spo0A* gene was amplified with the primers spoA166f and spoA748r, as previously described (Wunderlin et al., 2013). For quality filtering, the nucleotide sequences were translated to their amino acid sequences, based on ORF detection. The amino acid sequences were then aligned and compared to a Gribskov-style protein profile of Spo0A (Gribskov et al., 1987) that was built based on 27 known Spo0A sequences. Filtration was applied as a function of the profile score and profile alignment length, which separates noise or negatives hits from true positive *spo0A* sequences. The nucleotide sequences were clustered into operational taxonomic units (OTU) at 97% sequence identity using uclust (Edgar, 2010). The centroid (representative sequence) of each OTU was classified using MLgsc, a general sequence classifier adapted for protein sequence and customized to a Spo0A database (Junier et al., 2015).

All sequences were submitted to Sequence Read Archive of the National Center for Biotechnology Information (NCBI) under BioProject IDs PRJNA267761 and PRJNA276803.

### Statistical Analysis

Statistical analyses were performed using R, version 3.0.2 (R Core Team, 2013), Rstudio, version 0.98.1049, and BiodiversityR (Kindt and Coe, 2005). Correlations between diversity and environmental limiting factors were estimated using both Pearson’s and Spearman’s methods, however, since the data is not normally distributed, Spearman’s tests were considered as more appropriate and therefore applied to this dataset. Since we focused on inferring the effect of each environmental parameter on the relative GCN (*spo0A*/ 16S rRNA gene counts), a generalized additive model (GAM) was used to represent graphically the dependence of the relative GCN values to the environmental factors. Moreover, in order to determine the significance of the difference in the ratio obtained for three decision nodes pre-defined from the data (multiple, single and no limiting factor), ratios were log-transformed and the statistical significance was evaluated using a Kruskal-Wallis *post hoc* tests according to Nemenyi using the PMCMR (Pairwise Multiple Comparison of Mean Ranks Package) library in R, after verifying this model’s assumptions. This test was chosen, after performing a Shapiro-Wilk normality test (Multiple *p*-value = 0.047, Single *p*-value = 0.459, No Factors *p*-value = 0.102), which showed no normal distribution invalidating a classical ANOVA approach. All scripts used for the statistical analysis are provided as Supplementary Material.

## Results

### Characterization of the Natural Springs

In this study, 71 sampling points in 24 locations were investigated (**Supplementary Figure 1**). The samples were collected from geothermal springs and drillings (Chile: Salar de Aguas Calientes, Lirima wetland, El Tatio geysers; Greece: Eleftheres, Krinides, Lagadas, Milos, Nea Apollonia, Nigrita, Potamia, Pozar, Thermia, Traianoupoli; Germany: Gross Schoenebeck and Bruschal power plants; France: Soultz-sous-forets power plant; and Colombia: Los Volcanes) and mineral springs (Greece: Agia Paraskevi, Aggistro, Pikrolimni; Switzerland: Ponts-de-Martel). The sites studied exhibited diverse environmental characteristics concerning temperature, UV radiation, low and high pH, and mineral compounds present (**Supplementary Table 2**).

A large variation in DNA yield was observed based on the initial sample type (i.e., soil, sand, mud, sediment, biofilm, microbial mat, water; **Supplementary Figure 2**). This was particularly noticeable in water samples that had significantly lower biomass than soils, sediments, biofilms or microbial mats. Total bacterial abundance was determined by quantifying the 16S rRNA gene GCN, while EFF abundance was measured using GCN for the specific *spo0A* transcriptional factor gene. Relative GCN (*spo0A* GCN/16S rRNA gene GCN) ranged from <0.0001 to 100 %, with an average GCN of 6.79% (**Supplementary Table 3**). Considering that the 16S rRNA gene is found in multiple copies per bacterial genome (Farrelly et al., 1995), while *spo0A* gene is a single-copy gene (Galperin et al., 2012; Bueche et al., 2013), in addition to the comparison without normalization, a second calculation was made using for normalization the average number of rRNA gene operons found in Bacteria and in Firmicutes (Ratio % rrnDB; **Supplementary Table 3**). The normalization did not change the patterns of relative abundance obtained and thus further analyses were conducted using the non-normalized ratio.

### Single Environmental Factors Do Not Influence EFF Relative Abundance

**FIGURE 1:**
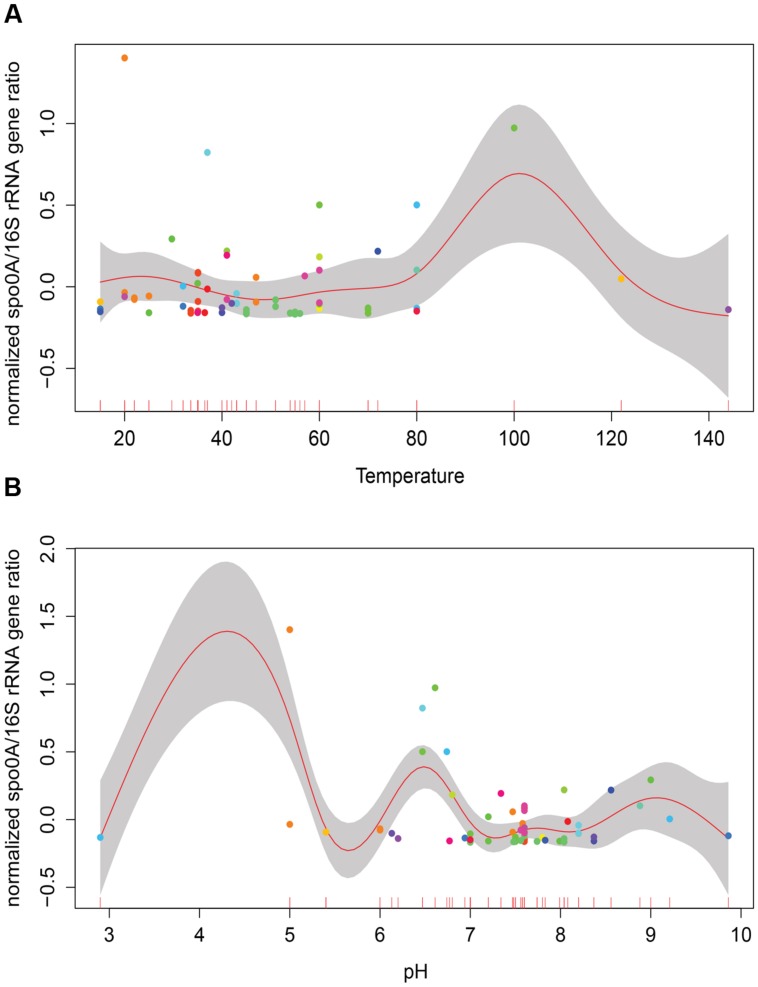
**Correlation of relative presence of EFF and selected environmental factors.** Generalized additive models depicting the relative presence of Endospore-forming Firmicutes –EFF- (*spo0A*/16S rRNA gene ratio; *y*-axis) to the *in situ* measurements of temperature **(A)**, pH **(B)**. No significant correlation between relative presence of EFF and these two factors is observed.

Temperature and pH are among factors that have been suggested to influence the abundance and diversity of microbial communities in geothermal sites (Hou et al., 2013; Sharp et al., 2014). Considering the variation in the number of samples within different categories (i.e., larger number of thermophilic versus mesophilic environments), a generalized additive model (GAM) was used to analyze the role of temperature and pH on relative GCN of EFF (**Figure 1**). No significant correlation between relative EFF GCN and temperature or pH was obtained (Temperature *R*^2^ = -0.233, *p*-value = 0.272; pH *R*^2^ = -0.144, *p-*value = 0.232).

### EFF Are Prevalent in Multiple-Limiting Environmental Factors

**FIGURE 2:**
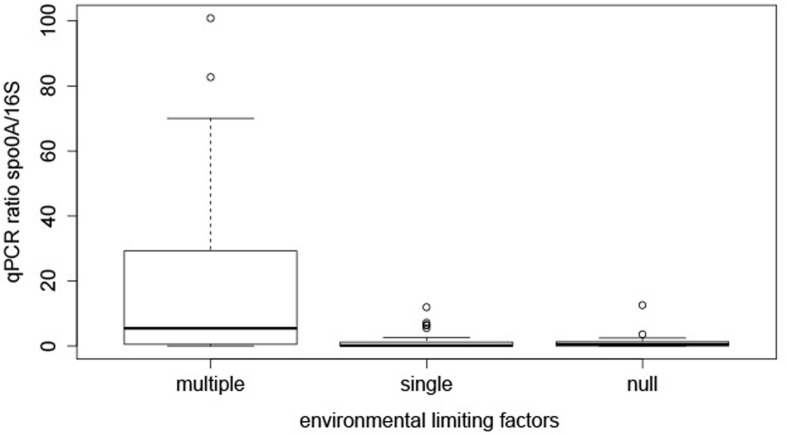
**Effect of multiple, single, and no limiting factors on relative presence of EFF.** The boxplots represent the qPCR ratios of *spo0A* gene/16S rRNA gene, grouped by decision node.

In order to try to find an explanation to the distribution of EFF in these springs the samples were grouped into three categories by transforming the quantitative measurements of limiting environmental factors into a qualitative limiting index. For the transformation we used as reference physicochemical parameters and ranges considered to describe mesophilic conditions (**Supplementary Table 1**). This included temperature (20 to 55°C), pH (5.5 to 8.5), atmospheric pressure (∼1 atm), exposure to UV radiation and concentration of cell-toxic chemical compounds. This categorization is far from perfect as the relative importance of individual factors might be different for different species and this is ignored when giving the same weight to each factor studied here. However, this scoring was selected because a large number of our samples are far from this mesophilic range, and thus using another approach (e.g., quintiles or percentiles) would result in the underestimation of limiting conditions in a traditional sense. Three categories of limiting environmental factors (multiple, single, or null) were defined. In total, 21, 28, and 22 samples from multiple, single and null limiting environments were assigned to each category. We next analyzed if the co-existence of multiple limiting factors could affect the relative abundance of EFF. Based on the multiple-single-null grouping, EFF had a statistically significant higher relative GCN (median ratio = 20.43%) in sites with multiple limiting environmental factors, compared to environments with single (median ratio = 1.56 %) or null (ratio median = 1.27%) factors (**Figure 2**). *Post hoc* tests showed that the difference between “multiple” and “single” factor groups was statistically significant (*p*-value = 0.0007), and so was between “multiple” and “null” groups (*p*-value = 0.006).

### Diversity of EFF in environmental samples

In order to evaluate the effect of limiting environmental factors on diversity, we analyzed the bacterial community composition of eight samples including representatives of the three categories shown in **Figure 2**. Firstly, we used data obtained from 16S rRNA gene amplicon pyrosequencing (**Table 1**). A total of 117,542 high quality sequence reads were obtained with 8,050–29,335 sequences per sample (mean 14,693). Sequence clustering at 97% identity resulted in 17,596 operational taxonomic units (OTUs) in the data set. Overall, richness in samples from sites with varying limiting environmental factors (null to multiple) remained stable and did not decrease with an increase in limiting conditions.

**Table 1:** Samples selected for diversity analysis.

Code	Location	Source	Extremity factor	*Spo0A*/16S rRNA Ratio %	Q-Reads	Total bacterial community				Firmicutes fraction			
							
						Shannon index	Simpson index	Richness - obs. OTUs (not rarefied)	OTUs/Chao1 %	Shannon index	Simpson index	Richness (not rarefied)	OTUs/Chao1 %
4nap-1	Nea Apollonia (GR)	Biofilm from drilling pipe	Multiple	100	17348 (436)	9.7	0.996	2490 (4452)	0.332	7.472	0.985	403 (1661)	0.284
Col	Los volcanos (CO)	Biofilm	Single	11.9	21886 (368)	5.6	0.889	900 (1877)	0.317	7.264	0.985	357 (1160)	0.302
49the-1	Thermia (GR)	Sediment	Single	1.18	11709 (121)	7.98	0.983	1353 (1770)	0.466	N/A	N/A	N/A (86)	N/A
51the-3	Thermia (GR)	Biofilm	Single	7.15	29335 (550)	6.524	0.932	1216 (3020)	0.351	7.057	0.969	400 (1290)	0.264
44agp-2	Agia Paraskevi (GR)	Precipitate	Null	0.06	10616 (14)	6.647	0.955	888 (1122)	0.38	N/A	N/A	N/A (97)	N/A
25kam-6	Kanava, Milos (GR)	Marine water	Null	0.41	9201 (31)	4.822	0.863	521 (596)	0.42	N/A	N/A	N/A (1)	N/A
NeFer	Ponts-de-Martel, iron (CH)	Water and sediment	Null	0.11	9397 (102)	9.781	0.995	2493 (2912)	0.41	5.895	0.917	264 (287)	0.443
Nesul	Ponts-de-Martel, sulfur (CH)	Water and sediment	Null	0.03	8050 (97)	7.976	0.978	1742 (1847)	0.374	N/A	N/A	N/A (194)	N/A


*In the Q-reads column the number in parenthesis corresponds to chimeras removed from further analysis. Values for the overall community correspond to samples rarefied to 7302 reads. Values for Firmicutes sub-community correspond to samples rarefied to 994 reads. (N/A = Not available).*

**FIGURE 3:**
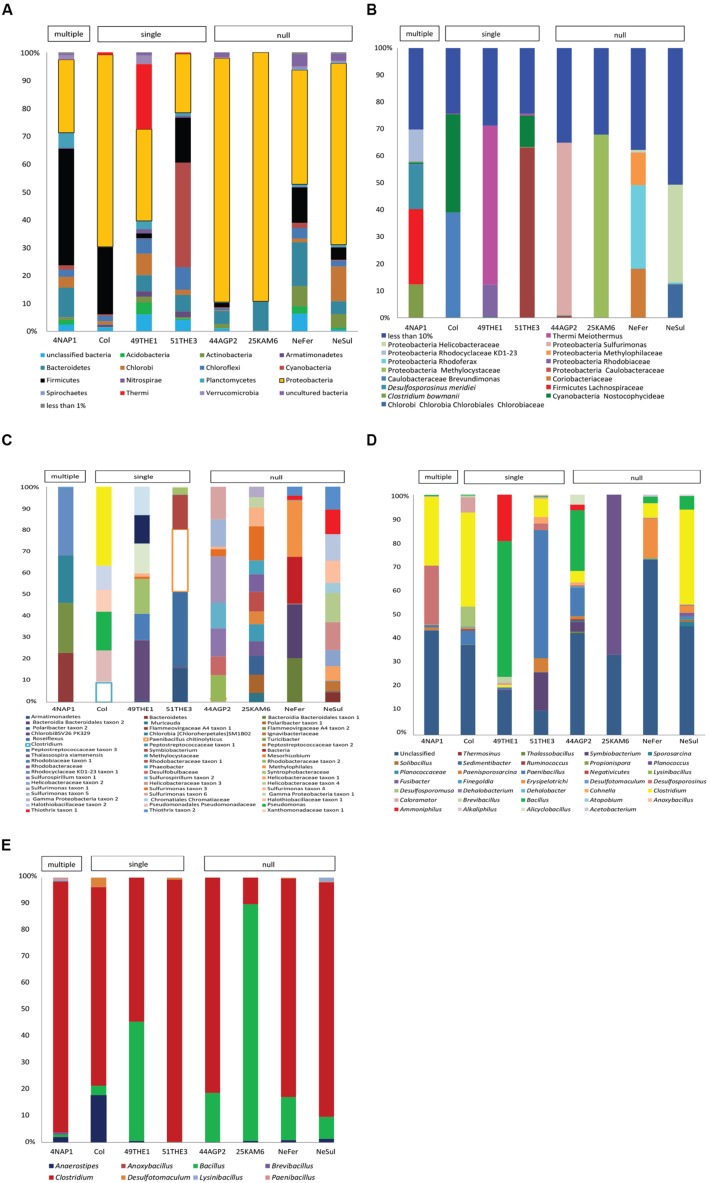
**Diversity analysis of eight samples: 4NAP1 (Nea Apollonia, Greece, multiple extreme environmental factors, biofilm, and water from thermal spring boring); Col, 49THE1 and 51THE3 (Los Volcanos, Colombia, biofilm and water from thermal spring; Thermia, sediment from hot spring, Thermia, sediment from mineral spring, respectively, single extreme environmental factor); 44AGP2, 25KAM5, NeFer and NeSul (Agia Paraskevi spring, sediment from the thermal spring; Kanava beach, Milos, Greece, water, and sand from the beach shore; Ponts-de-Martel water, biofilm and precipitates from the Iron and Sulfur springs, Switzerland, respectively, no extremity factor present).** **(A)** Total community composition based on 16S rRNA gene sequencing data to phylum level. **(B)** Total community composition represented by the most abundant OTUs (more than 10%). **(C)** The fraction of the low frequency OTUs of the total community. **(D)** Community composition of Firmicutes, based on 16S rRNA gene sequencing data. **(E)** Community composition based on *spo0A* gene sequencing data. OTUs detected were classified to known endospore-forming genera.

Firmicutes represented a significant fraction of the community in some samples (**Supplementary Figure 3**), regardless of the type of environmental conditions of the site. However, it is important to indicate that the identification of endospore-formers based on the 16S rRNA gene is not entirely possible because of the patchiness of the distribution of sporulation as a trait within related Firmicute clades. Total community compositions at a phylum level are represented in **Figure 3A**. Considering the total community composition at this taxonomic level, the most prevalent phylum in the sample influenced by multiple extreme environmental parameters, temperature, uranium and alkaline pH (4NAP1), was Firmicutes (41,7%), followed by Proteobacteria (26,14 %) and Bacteroidetes (10.54%). In the samples influenced by a single extreme environmental factor, Firmicutes were less abundant. In a thermal spring in Los Volcanos, Colombia (Col), the most abundant phylum was Proteobacteria (68.85%), followed by Firmicutes (23.97%). Two other single factor samples were analyzed at a community composition level. These two samples were collected from two different springs at close proximity from Thermia, Greece. The one spring (49THE1) is a thermal spring used for bathing purposes, while the second one (51THE3) is a mineral spring exposed to sunlight. The community composition between these two sites varied significantly, with the thermal spring being mostly dominated by Proteobacteria (32.81%), followed by Thermi (23.38%), while the spring exposed to sunlight was mostly dominated by Cyanobacteria (37.41%), followed by Proteobacteria (21.03%) and Firmicutes (16.1%). The dominance of Cyanobacteria in this spring is explained by the sampling period (summertime). At the sampling sites where no extreme environmental parameter was measured, bacterial communities were mostly dominated by Proteobacteria and Firmicutes represented only a small fraction. When considering the most abundant OTUs in the total bacterial communities, these clearly belong to Proteobacteria, followed by Firmicutes (**Figure 3B**). The low frequency groups (less than 10%) showed a high diversity, and in some cases represented up to 50% of the total abundance, revealing that these groups are either able to co-exist with the most prevalent taxa, or that they can serve as seed banks for future micro-environmental changes in the communities, such as in the case of *Clostridium* sp., *Paenibacillus chitinolyticus* and *Turicibacter* sp. (**Figure 3C**). Focusing on the fraction of Firmicutes in the total communities (**Figure 3D**), we observed a large fraction of reads that could not be classified into OTUs (unclassified Firmicutes). The remaining reads were mostly classified into OTUs identified as *Clostridium* and *Bacillus*. Finally, in springs used for bathing purposes (Colombia, Greece), species belonging to *Lachnospiraceae* were found to be abundant, an observation that agrees with the human origin of this family (Meehan and Beiko, 2014).

The endospore-forming group of Firmicutes was also studied. Amplicon sequencing of the *spo0A* gene resulted in a total of 14,362 quality reads with an average length of 491 bp. These reads were clustered into 3,392 OTUs, applying a 97% identity threshold. The OTUs were assigned to eight different genera (**Supplementary Table 4**). In the community composition analysis, three genera were common to all the samples: *Clostridium. Bacillus* and *Anaerostipes. Clostridium* was the most prevalent genus, with the exception of 25KAM6 that was dominated by *Bacillus* (**Figure 3E**).

## Discussion

It has been suggested that because of their dispersal potential and diverse metabolic capabilities, endospore-forming Firmicutes (EFF) are one of the most ubiquitous microbial groups (Roberts and Cohan, 1995; Marchant et al., 2002, 2008; Martiny et al., 2006). Interestingly, in some cases, EFF are not only present, but also in high numbers, suggesting that EFF are not only ubiquitous but they can also persist in habitats that contradict their lifestyle, as for example shown in a study of isolation of endemic thermophilic EFF in cool soils (Marchant et al., 2002). However, there is no conclusive experimental evidence showing this. On the contrary, many molecular ecology diversity surveys have failed to detect this group (von Mering et al., 2007; Filippidou et al., 2015). Our results offer, for the first time, experimental evidence regarding the ubiquity of this group of Firmicutes in mineral springs. We detected a variable GCN of EFF in the majority of environments studied. However, for most of the sites, EFF represent only a small fraction of the total bacteria observed in the samples, probably explaining the previous difficulties of detecting this rare component of the microbial community.

An ecological explanation to the poor representation of EFF relative to the total bacterial community can be found when analyzing the tradeoffs of the survival strategies deployed by EFF. This is particularly clear for endospore-formation, a notably energy-demanding process (Höfler et al., 2016) that leads to the formation of a resistance structure. EFF have been used as models to investigate the cost of relative simple phenotypes (e.g., maintenance of simple biosynthetic pathways; Maughan et al., 2007), as well as more complex ones, such as spore development (Callahan et al., 2008). An experimental evolution experiment with populations of *B. subtilis* with and without a selection for spore development has shown that loss of sporulation could affect positively the energetic flux and growth rate of this bacterium, and thus is selected against by simply mutational degradation under non-selective environmental conditions (Maughan et al., 2007). Although the energy requirements of other survival strategies deployed by EFF are not known, it is possible to consider that the biological cost of their myriad of survival strategies limits the distribution of this group under non-limiting conditions. This was the hypothesis tested in this study. However, the definition of an “extreme” factor that limits microbial growth is not easy as “*extreme is in the eyes of the beholder”* (Rothschild and Mancinelli, 2001). Upper limits to life have been suggested (Harrison et al., 2013; Cockell et al., 2016), as well as upper limits of habitable ecosystems (Cockell, 2015). In geothermal and mineral springs a combination of steep physicochemical gradients might offer EFF an advantageous niche. First, the availability of dissolved organic carbon in the water is limited (Edwards, 1990). Second, in terrestrial springs compared to marine hydrothermal vents, molecular oxygen is either absent or in low concentrations (Reysenbach and Shock, 2002). Finally, hotspots of geothermal springs exhibit temperature exceeding 100°C and are most of the times highly acidic (Hou et al., 2013). A gradient of temperature, pH and dissolved minerals is formed (Weltzer and Miller, 2013), creating a gradient of habitats and niches because of the different ecological optima of various taxa.

The results obtained here support our hypothesis suggesting that limiting environmental conditions favor the relative abundance of EFF. However, this was true only when the data were analyzed by regrouping the samples to consider the co-existence of various limiting factors. Theoretically, a single limiting factor should suffice to reduce total bacterial abundance and consequently to result in a relative enrichment of endospore-formers. However, our data show that there is no significant difference between environments with no limiting factors and others where a single limiting factor is present. This indicates that even though each individual limiting factor studied here is reported to reduce microbial abundance in general (Losick and Stragier, 1992; Errington, 1993; McDougald et al., 2002; Nicholson et al., 2002; Setlow et al., 2002; Sanchez-Salas et al., 2011; Müller et al., 2014), this by itself does not result in an increase in the relative abundance of EFF. Our data also suggest that abundance, species richness, and diversity do not depend on the limiting factor. It is the co-existence of limiting factors that apparently drives the increase in prevalence of EFF in the environments studied here.

Studies analyzing the role of environmental factors in distribution patterns of microbial communities in environments with limiting environmental conditions have started to emerge. For instance, in geothermal environments, recent publications have shown that temperature (Sharp et al., 2014), and, to a lesser extent, pH (Hou et al., 2013), dictate the prevalence of specific bacterial and archaeal groups. In other environments, such as salt flats (salars), salinity is believed to control microbial distribution (Oren, 2001). These environmental factors determine the distribution of individuals, but they can also explain the distribution of a population or even a community (Woodward, 1987; Calow and Sibly, 1990; Pörtner, 2002). Although one could argue that not all environmental factors determine to the same extent the ecological niche of a species, which is most likely the case in nature, a general theoretical unimodal distribution model with maximum abundance toward the middle range of individual environmental factors has been predicted based on Shelford’s law of tolerance, according to which “[an organism] is absent or found in minimal numbers only […] should a [environmental] condition vary outside the limits tolerated by the animal” (Shelford, 1931). So far, the same model has been applied for diversity. It has often been discussed that in the case of microbial communities, abundance and diversity also decrease towards extremity in the case of temperature and pH (Oren, 2001; Fierer and Jackson, 2006), possibly following Shelford’s law. This has been supported by patterns of species distribution across altitudinal gradients for different taxa such as *Acidobacteria* (Lozupone and Knight, 2007; Bryant et al., 2008).

Based on our results, EFF GCN and diversity do not follow a theoretical unimodal model of abundance and diversity, at least in the case of geothermal and mineral springs. In fact, if strategies such as dormancy are in place, limiting environmental conditions may play a subtler role on community structure because total community composition may differ largely from active populations. It has been proposed that EFF persist in the environment primarily in a spore state rather than as vegetative cells (Zeigler, 2013), which could explain the distribution pattern observed here. For example, the co-existence of active and dormant Firmicutes, can be supposed in the case of the 16S rRNA libraries from the 4NAP1 sample in which *Clostridium bowmanii* and *Desulfosporosinus meridiei* are two prevalent species among Firmicutes. Both species are anaerobic, but the former is considered as a psychrophile and the later a sulfur-reducing bacterium, also involved in uranium bioremediation (Nevin et al., 2003). Considering that the thermal spring of Nea Apollonia is highly thermophilic, the prevalence of *C. bowmanii* could be explained by its presence as endospores. On the other hand, the presence of *D. meridiei* can be explained by the uranium contamination of the spring (i.e., potentially active). In aquatic ecosystems, high water temperature would lead to a decrease in the concentration of dissolved oxygen, which may result in anoxic conditions that favor anaerobic species (such as clostridia) to thrive. As far as our *spo0A* libraries are concerned, the strictly anaerobic *Clostridium* sp. dominated community composition in all the samples. Five out of eight of the sites sequenced, corresponded to high temperature springs. However, for two of the other mineral springs (NeSul and NeFer), *Clostridium* is still prevalent even though the temperature on the site was 15°C. The dominance of anaerobic Firmicutes suggests once more the idea that these bacteria are found in the state of endospores in these sites, remaining dormant until conditions change or dispersal is possible (seed banks). Although additional experiments to measure the numbers of spores in each environment are needed to evaluate the role of sporulation, the results obtained in our study suggest that in the environment, multiple factors would be operationally significant to act as sporulation triggers. In fact, the environmental triggers of sporulation are still unclear, and even among closely related strains, there is no unique trigger (Setlow, 2006; Duc et al., 2007; Mohapatra and La Duc, 2012; Mohapatra and Duc, 2013).

## Conclusion

The results of relative abundance and diversity obtained here suggest that commonly used patterns of bacterial distribution cannot be applied to EFF. In addition, the fact that we observed an enrichment of EFF in multiple limiting factors but not in response to single ones suggests that the effects of limiting environmental conditions are not additive, but rather multiplicative. This observation has a profound effect in our ability to predict EFF distribution in natural environments.

## Author Contributions

PJ designed the research. SF and TW conducted the experiments and statistical analysis. DJ contributed to statistical analysis. TJ performed bioinformatics analysis. SF, TW, NJ, CD, VM, and PJ held the sampling. SF and PJ wrote the manuscript. All authors contributed to the interpretation of data and to the critical revision of the manuscript.

## Conflict of Interest Statement

The authors declare that the research was conducted in the absence of any commercial or financial relationships that could be construed as a potential conflict of interest.

## Footnotes

1 https://rdp.cme.msu.edu/tutorials/aligner/RDPtutorial_ALIGNER.html
