# Supplementary material for: A Combination of Extreme Environmental Conditions Favor the Prevalence of Endospore-Forming Firmicutes
Source: Front Microbiol. 2016 Nov 3;7:1707. doi: 10.3389/fmicb.2016.01707 (PMC5094177; doi:10.3389/fmicb.2016.01707)
Supplement: Supplementary file 1 [file Data_Sheet_1.docx]

Supplementary Material

A combination of extreme environmental conditions favor the prevalence of Endospore-forming Firmicutes

**Sevasti Filippidou, Tina Wunderlin, Thomas Junier, Nicole Jeanneret, Cristina Dorador, Veronica Molina, David R. Johnson, Pilar Junier^*^**

*** Correspondence:** Corresponding Author: Prof. Pilar Junier [pilar.junier@unine.ch](mailto:pilar.junier@unine.ch)

# Supplementary Figures and Tables

## Supplementary Table 1

| Supplementary Table 1. Theoretical information on abiotic environmental factors that influence niche differentiation. Ranges and description of limiting conditions are restricted to Bacteria and Archaea. The role of those factors in sporulation of endospore-forming Firmicutes is also indicated. *In the column “Habitat” only those used for sampling in this study are mentioned. Numerous other environments can have the below mentioned characteristics but are not indicated here. | | | | | | |
| --- | --- | --- | --- | --- | --- | --- |
| Factor | **Subdivision** | **Definition** | **Range** | **Limiting** | **Sporulation** | **Habitat*** |
| Temperature | Psychrophilic | As a result of Earth’s tilt towards the sun and altitude (Molles, 2005) | -20 to 12°C (Barton and Northup, 2011; Madigan et al., 2010) | Yes except psychrophiles | Yes (Müller et al., 2014) | Lake bottom and sediments |
|  | Thermophilic | “As a result of volcanic activity or movement of the Earth’s crust at tectonically active sites” (Edwards, 1990) | 55 to 130°C (Edwards, 1990; Kashefi and Lovley, 2003) | Yes except thermophiles and thermotolerant | Yes (Nicholson et al., 2002; Sanchez-Salas et al., 2011) | Hot springs,  Geothermal drillings |
| pH | Acidic | “Acid environments that provide the thermodynamic potential for cell maintenance and growth” (Edwards, 1990) | 1 to 4  (Edwards, 1990; Madigan et al., 2010) | Yes | Yes (Setlow et al., 2002) | Hot springs,  Mineral springs,  Geothermal drillings |
|  | Alkaline | “Proton concentration low, but also low availability of other metabolites and ions that precipitate (insoluble)” (Edwards, 1990) | 8.5 to 12 (Madigan et al., 2010) | Yes | Yes (Setlow et al., 2002) | Hot springs,  Mineral springs,  Salars |
| Light^&^ | UV light | Fluxes of solar ultraviolet radiation may be a pervasive and potentially damaging influence for both aquatic and terrestrial ecosystems (e.g. Karentz, 1994; Zagarese and Williamson, 1994; Diffey, 1991) | (UVR, 280-400nm) | Yes, DNA damage (Barton and Northup, 2011; Morris et al., 1995) | Yes (Nicholson et al., 2000; Sanchez-Salas et al., 2011) | Atakama Dessert |
| Salinity | Halophilic | As a result of high rates of evaporation resulting in desiccation (Edwards, 1990) | 6-32% (w/v) (Madigan et al., 2010) | (indirectly) desiccation | Yes (Nicholson et al, 2000), for desiccation) | Salars |
| Pressure | barophilic | high hydrostatic pressures encounted in deep sea and reservoir habitats (Barton and Northup, 2011) | 500 to 1000 atm  (Madigan et al., 2010) |  | Higher than 200 atm (Nicholson et al., 2000) | Geothermal drillings |

## Supplementary table 2: Main Mineral Composition analysis performed by: *IGME, Institute for Geological Research in Greece, published in 2009, Athens; &= Mineral Composition not determined (N/D) or not applicable (N/A) for these samples; # Measured for this research i*n situ*.

| **Supplementary Table 2 :** **Sampling sites, environmental factors and extremity index** | | | | | | | | | |
| --- | --- | --- | --- | --- | --- | --- | --- | --- | --- |
| **Location** | **Sample ID** | **Sample type** | **Tempe-rature** | **pH** | **Main Mineral composition** | T-quali | pH-quali | Radiation | Extremity |
| **Aggistro*** | 13agg1 | Water | 36.46 | 7 | Ca-Na- HCO3 | 0 | 0 | 0 | 0 |
| **Aggistro*** | 14agg2 | Biofilm | 37 | 8.08 | Ca-Na- HCO4 | 0 | 0 | 0 | 0 |
| **Agia Paraskevi*** | 43AGP-1 | Water | 33.59 | 7.6 | H2S, CO2 | 0 | 0 | 0 | 0 |
| **Agia Paraskevi*** | 44AGP-2 | Precipitate | 33.59 | 7.6 | H2S, CO2 | 0 | 0 | 0 | 0 |
| **Agia Paraskevi*** | 45AGP-3 | Precipitate | 35.07 | 7.6 | H2S, CO2 | 0 | 0 | 1 | 1 |
| **Agia Paraskevi*** | 46AGP-4 | Precipitate | 35.07 | 7.6 | H2S, CO2 | 0 | 0 | 1 | 1 |
| **Agia Paraskevi*** | 47AGP-5 | Precipitate | 35.07 | 7.6 | H2S, CO2 | 0 | 0 | 1 | 1 |
| **Agia Paraskevi*** | 48AGP-6 | Biofilm | 35.07 | 7.6 | H2S, CO2 | 0 | 0 | 1 | 1 |
| **Aguas Calientes&** | Ac3b | Sediment | 22 | 6 | N/D | 0 | 0 | 0 | 0 |
| **Aguas Calientes&** | Ac5b | Sediment | 25 | 7.58 | N/D | 0 | 0 | 0 | 0 |
| **Aguas Calientes&** | Ac6b | Sediment | 47 | 7.47 | N/D | 0 | 0 | 0 | 0 |
| **Aguas Calientes&** | Ac3a | Sediment | 22 | 6 | N/D | 0 | 0 | 1 | 1 |
| **Aguas Calientes&** | Ac5a | Sediment | 25 | 7.58 | N/D | 0 | 0 | 1 | 1 |
| **Aguas Calientes&** | Ac6a | Sediment | 47 | 7.47 | N/D | 0 | 0 | 1 | 1 |
| **Aguas Calientes&** | Ac4b | Sediment | 20 | 5 | N/D | 0 | 1 | 0 | 1 |
| **Aguas Calientes&** | Ac4a | Sediment | 20 | 5 | N/D | 0 | 1 | 1 | 2 |
| **Bruschal#** | **Br2** | **Water** | **122** | **5.4** | **Ca-Mg-Na-K-Sr-Rb** | **1** | **1** | **0** | **3** |
| **Bruschal#** | **Br3** | **Water** | **122** | **5.4** | **Ca-Mg-Na-K-Sr-Rb** | **1** | **1** | **0** | **3** |
| **Bruschal#** | **Br6** | **Water** | **122** | **5.4** | **Ca-Mg-Na-K-Sr-Rb** | **1** | **1** | **0** | **3** |
| **Bruschal#** | **Br7** | **Biofilm** | **15** | **5.4** | **Ca-Mg-Na-K-Sr-Rb** | **1** | **1** | **0** | **3** |
| **Charos Adama** | 28cam1 | Water | 60 | 7.8 | N/D | 1 | 0 | 0 | 1 |
| **Colombia** | Col | Biofilm | 60 | 6.8 | N/A | 1 | 0 | 0 | 1 |
| **Eleftheres*** | 12ele1 | Soil | 41 | 8.04 | Na-HCO3-Cl-Ca | 0 | 0 | 0 | 0 |
| **Kanava, Milos*** | 20kam-1 | Sand | 70 | 7.5 | H2S, CO2 | 1 | 0 | 1 | 2 |
| **Kanava, Milos*** | 21kam-2 | Sand | 70 | 7.5 | H2S, CO3 | 1 | 0 | 1 | 2 |
| **Kanava, Milos*** | 22kam-3 | Sand | 70 | 7.5 | H2S, CO4 | 1 | 0 | 1 | 2 |
| **Kanava, Milos*** | 23kam-4 | Sand | 100 | 6.61 | H2S, CO5 | 1 | 0 | 1 | 2 |
| **Kanava, Milos*** | 24kam-5 | Water | 60 | 6.47 | H2S, CO6 | 1 | 0 | 1 | 2 |
| **Kanava, Milos*** | 25kam-6 | Water | 60 | 7 | H2S, CO7 | 1 | 0 | 1 | 2 |
| **Krinides*** | 10kri2 | Water | 25 | 7.99 | H2S, CO8 | 0 | 0 | 0 | 0 |
| **Krinides*** | 9kri1 | Water | 29.7 | 9 | H2S, CO9 | 0 | 1 | 1 | 2 |
| **liogerma** | 31alm1 | Water | 35 | 7.2 | N/D | 0 | 0 | 0 | 0 |
| **liogerma** | 32alm2 | Biofilm | 35 | 7.2 | N/A | 0 | 0 | 0 | 0 |
| **Lirima&** | Lr10 | Microbial mat | 45 | 8.04 | N/A | 0 | 0 | 1 | 1 |
| **Lirima&** | Lr11 | Microbial mat | 45 | 8.04 | N/A | 0 | 0 | 1 | 1 |
| **Lirima&** | Lr12 | Microbial mat | 45 | 8.04 | N/A | 0 | 0 | 1 | 1 |
| **Lirima&** | Lr13 | Microbial mat | 45 | 8.04 | N/A | 0 | 0 | 1 | 1 |
| **Lirima&** | Lr2 | Microbial mat | 54 | 7.74 | N/A | 0 | 0 | 1 | 1 |
| **Lirima&** | Lr3 | Microbial mat | 54 | 7.74 | N/A | 0 | 0 | 1 | 1 |
| **Lirima&** | Lr5 | Microbial mat | 45 | 7 | N/A | 0 | 0 | 1 | 1 |
| **Lirima&** | Lr6 | Microbial mat | 51 | 7.6 | N/A | 0 | 0 | 1 | 1 |
| **Lirima&** | Lr7 | Microbial mat | 51 | 7.6 | N/A | 0 | 0 | 1 | 1 |
| **Lirima&** | Lr1 | Microbial mat | 56 | 7.48 | N/A | 1 | 0 | 1 | 2 |
| **Lirima&** | Lr8 | Microbial mat | 55 | 7.56 | N/A | 1 | 0 | 1 | 2 |
| **Lirima&** | Lr9 | Microbial mat | 55 | 7 | N/A | 1 | 0 | 1 | 2 |
| **Nea Apollonia*#** | 5nap-2 | Biofilm | 80 | 8.2 | Na-HCO3-SO4 | 1 | 0 | 0 | 1 |
| **Nea Apollonia*#** | **4nap-1** | **Biofilm** | **80** | **8.88** | **Na-HCO3-SO4-U** | **1** | **1** | **0** | **3** |
| **Nigrita*** | 39NIG-1 | Soil | 43 | 8.2 | Mg-Ca-Na-HCO3 | 0 | 0 | 0 | 0 |
| **Nigrita*** | 40NIG-2 | Sediment | 43 | 8.2 | Mg-Ca-Na-HCO4 | 0 | 0 | 0 | 0 |
| **Nigrita*** | 41NIG-3 | Sediment | 43 | 8.2 | Mg-Ca-Na-HCO5 | 0 | 0 | 0 | 0 |
| **Palaiochori** | 17pam2 | Rock | 37 | 6.47 | N/A | 0 | 0 | 0 | 0 |
| **Palaiochori** | 16pam1 | Sediment | 80 | 2.9 | N/A | 1 | 0 | 1 | 2 |
| **Palaiochori** | 18pam3 | Sand | 80 | 6.74 | N/A | 1 | 0 | 1 | 2 |
| **Pikrolimni*** | 2pik1 | Sediment | 32 | 9.21 | H2S, CO2 | 0 | 1 | 1 | 2 |
| **Pikrolimni*** | 3pik2 | Sediment | 32 | 9.86 | H2S, CO3 | 0 | 1 | 1 | 2 |
| **Ponts-de-Martel, Iron** | NeFer | Sediment | 15 | 6.94 | N/D | 1 | 0 | 0 | 1 |
| **Ponts-de-Martel, Sulfur** | NeSulf | Sediment | 15 | 7.83 | N/D | 1 | 0 | 0 | 1 |
| **Potamia*** | 8pot-1 | Biofilm | 72 | 8.56 | Na-Cl-HCO3 | 1 | 1 | 0 | 2 |
| **Pozar*** | 36POZ-2 | Aggregates | 40 | 8.37 | Ca-Mg-HCO3 | 0 | 0 | 0 | 0 |
| **Pozar*** | 37POZ-3 | Biofilm | 40 | 8.37 | Ca-Mg-HCO4 | 0 | 0 | 0 | 0 |
| **Provatas** | 15prm1 | Biofilm | 42 | 6.13 | N/A | 0 | 0 | 0 | 0 |
| **Soultz** | S3 | Water | 144 | 6.2 | N/D | 1 | 0 | 0 | 1 |
| **Thermia*** | 52the-4 | Sediment | 20 | 7.6 | Na-Ca-HCO3-SO4 | 0 | 0 | 0 | 0 |
| **Thermia*** | 49the-1 | Sediment | 57 | 7.6 | Na-Ca-HCO3-SO5 | 1 | 0 | 0 | 1 |
| **Thermia*** | 50the-2 | Biofilm | 60 | 7.6 | Na-Ca-HCO3-SO6 | 1 | 0 | 0 | 1 |
| **Thermia*** | 51the-3 | Water | 60 | 7.6 | Na-Ca-HCO3-SO7 | 1 | 0 | 0 | 1 |
| **Traianoupoli*** | 6tra1 | Biofilm | 41 | 7.56 | Na-Cl | 0 | 0 | 0 | 0 |
| **Traianoupoli*** | 7tra2 | Soil | 41 | 7.34 | Na-Cl | 0 | 0 | 0 | 0 |
| **Tria Pigadia** | 27tpm2 | Rock | 35 | 6.77 | N/A | 0 | 0 | 0 | 0 |
| **Zefuria plain** | 33zpm1 | Aggregates | 80 | 7 | N/D | 1 | 0 | 0 | 1 |
| **Zefuria plain** | 34zpm2 | Aggregates | 80 | 7 | N/D | 1 | 0 | 0 | 1 |

## Supplementary table 3

| Supplementary Table 3. Samples and normalized counts for 16S rRNA and spo0A gene copy numbers. The ratio between 16S rRNA and spo0A counts is presented in the last column (Ratio %). | | | | | | |
| --- | --- | --- | --- | --- | --- | --- |
| Location | Sample ID | 16S rRNA copy numbers | spo0A copy numbers | Ratio % | Ratio % (rrnDB) | Extremity Factor |
| Aguas Calientes | Ac6b | 1,90E+05 | 3,38E+03 | 1,7795 | 7,4026 | multiple |
| Bruschal | Br7 | 6,09E+07 | 3,59E+05 | 0,5895 | 2,4523 | multiple |
| Bruschal | Br3 | 9,34E+04 | 2,18E+03 | 2,3340 | 9,7096 | multiple |
| Bruschal | Br2 | 2,50E+03 | 1,17E+02 | 4,6800 | 19,4688 | multiple |
| Bruschal | Br6 | 4,00E+03 | 2,32E+02 | 5,8000 | 24,1280 | multiple |
| Kanava, Milos | 22kam-3 | 2,25E+06 | 1,09E+02 | 0,0049 | 0,0202 | multiple |
| Kanava, Milos | 20kam-1 | 8,03E+05 | 6,32E+02 | 0,0787 | 0,3275 | multiple |
| Kanava, Milos | 21kam-2 | 1,83E+06 | 2,83E+03 | 0,1546 | 0,6433 | multiple |
| Kanava, Milos | 25kam-6 | 8,44E+04 | 3,46E+02 | 0,4100 | 1,7054 | multiple |
| Kanava, Milos | 24kam-5 | 2,00E+06 | 7,72E+05 | 38,6000 | 160,5760 | multiple |
| Kanava, Milos | 23kam-4 | 5,03E+05 | 4,16E+05 | 82,7038 | 344,0477 | multiple |
| Krinides | 9kri1 | 2,15E+03 | 4,27E+02 | 19,8666 | 82,6450 | multiple |
| Lirima | Lr9 | 4,36E+06 | 8,36E+00 | 0,0002 | 0,0008 | multiple |
| Lirima | Lr7 | 8,02E+06 | 2,13E+02 | 0,0027 | 0,0110 | multiple |
| Lirima | Lr8 | 3,66E+06 | 7,28E+02 | 0,0199 | 0,0827 | multiple |
| Nea Apollonia | 4nap-1 | 1,86E+06 | 1,88E+06 | 100,8602 | 419,5785 | multiple |
| Palaiochori | 17pam2 | 5,26E+06 | 7,35E+03 | 0,1397 | 0,5810 | multiple |
| Palaiochori | 18pam3 | 9,61E+02 | 3,71E+02 | 38,5976 | 160,5660 | multiple |
| Pikrolimni | 3pik2 | 1,27E+06 | 3,13E+03 | 0,2464 | 1,0252 | multiple |
| Pikrolimni | 2pik1 | 3,46E+06 | 1,03E+05 | 2,9829 | 12,4090 | multiple |
| Potamia | 8pot-1 | 2,43E+06 | 3,46E+05 | 14,2222 | 59,1644 | multiple |
| Agia Paraskevi | 48AGP-6 | 1,80E+08 | 7,47E+04 | 0,0415 | 0,1725 | single |
| Agia Paraskevi | 46AGP-4 | 2,76E+07 | 1,72E+05 | 0,6229 | 2,5911 | single |
| Agia Paraskevi | 45AGP-3 | 4,00E+06 | 2,51E+05 | 6,2771 | 26,1128 | single |
| Agia Paraskevi | 47AGP-5 | 3,93E+06 | 2,53E+05 | 6,4408 | 26,7938 | single |
| Aguas Calientes | Ac5b | 1,39E+05 | 7,96E+02 | 0,5718 | 2,3787 | single |
| Aguas Calientes | Ac4b | 1,85E+05 | 1,55E+03 | 0,8387 | 3,4890 | single |
| Aguas Calientes | Ac5a | 6,15E+04 | 1,20E+03 | 1,9559 | 8,1367 | single |
| Aguas Calientes | Ac6a | 2,05E+05 | 5,11E+03 | 2,4948 | 10,3784 | single |
| Charos Adama | 28cam1 | 9,23E+02 | 1,26E+00 | 0,1365 | 0,5677 | single |
| Colombia | Col | 3,89E+07 | 4,63E+06 | 11,9023 | 49,5136 | single |
| Lirima | Lr1 | 5,46E+06 | 5,08E+01 | 0,0009 | 0,0039 | single |
| Lirima | Lr10 | 9,00E+06 | 2,19E+02 | 0,0024 | 0,0101 | single |
| Lirima | Lr2 | 3,90E+06 | 2,28E+02 | 0,0058 | 0,0243 | single |
| Lirima | Lr13 | 4,09E+06 | 2,93E+02 | 0,0072 | 0,0298 | single |
| Lirima | Lr3 | 7,29E+06 | 1,07E+03 | 0,0147 | 0,0611 | single |
| Lirima | Lr12 | 4,19E+06 | 3,04E+03 | 0,0726 | 0,3018 | single |
| Lirima | Lr11 | 2,21E+06 | 1,81E+03 | 0,0819 | 0,3407 | single |
| Lirima | Lr6 | 1,13E+07 | 2,60E+04 | 0,2299 | 0,9564 | single |
| Lirima | Lr5 | 7,17E+06 | 5,68E+04 | 0,7922 | 3,2955 | single |
| Nea Apollonia | 5nap-2 | 9,50E+05 | 6,83E+04 | 7,1895 | 29,9082 | single |
| Ponts-de-Martel, Iron | NeFer | 1,09E+07 | 1,20E+04 | 0,1095 | 0,4553 | single |
| Ponts-de-Martel, Sulfur | NeSulf | 7,27E+07 | 1,98E+04 | 0,0272 | 0,1132 | single |
| Soultz | S3 | 4,48E+07 | 3,85E+04 | 0,0859 | 0,3575 | single |
| Thermia | 52the-4 | 4,28E+05 | 2,17E+03 | 0,5061 | 2,1053 | single |
| Thermia | 50the-2 | 1,49E+06 | 8,11E+04 | 5,4430 | 22,6427 | single |
| Thermia | 51the-3 | 5,19E+07 | 3,71E+06 | 7,1484 | 29,7372 | single |
| Zefuria plain | 33zpm1 | 8,09E+05 | 3,33E+02 | 0,0411 | 0,1709 | single |
| Zefuria plain | 34zpm2 | 1,24E+06 | 8,44E+02 | 0,0683 | 0,2841 | single |
| Aggistro | 13agg1 | 1,56E+06 | 1,72E+02 | 0,0111 | 0,0461 | null |
| Aggistro | 14agg2 | 3,13E+07 | 7,47E+05 | 2,3899 | 9,9419 | null |
| Agia Paraskevi | 43AGP-1 | 9,88E+06 | 5,31E+02 | 0,0054 | 0,0224 | null |
| Agia Paraskevi | 44AGP-2 | 6,65E+06 | 3,91E+03 | 0,0589 | 0,2448 | null |
| Aguas Calientes | Ac3a | 4,61E+05 | 4,69E+03 | 1,0180 | 4,2350 | null |
| Aguas Calientes | Ac3b | 4,41E+05 | 5,54E+03 | 1,2552 | 5,2215 | null |
| Aguas Calientes | Ac4a | 1,05E+05 | 5,29E+03 | 5,0517 | 21,0151 | null |
| Eleftheres | 12ele1 | 6,22E+06 | 8,92E+05 | 14,3316 | 59,6195 | null |
| Krinides | 10kri2 | 1,80E+04 | 1,76E+00 | 0,0098 | 0,0407 | null |
| liogerma | 32alm2 | 2,18E+06 | 2,50E+02 | 0,0115 | 0,0478 | null |
| liogerma | 31alm1 | 3,68E+02 | 1,31E+01 | 3,5598 | 14,8087 | null |
| Nigrita | 39NIG-1 | 3,82E+06 | 1,69E+04 | 0,4420 | 1,8388 | null |
| Nigrita | 40NIG-2 | 4,26E+06 | 2,02E+04 | 0,4730 | 1,9678 | null |
| Nigrita | 41NIG-3 | 5,85E+05 | 9,39E+03 | 1,6042 | 6,6735 | null |
| Palaiochori | 16pam1 | 1,40E+03 | 9,79E+02 | 70,0587 | 291,4441 | null |
| Pozar | 36POZ-2 | 2,86E+06 | 3,38E+02 | 0,0118 | 0,0492 | null |
| Pozar | 37POZ-3 | 9,64E+05 | 1,59E+03 | 0,1646 | 0,6849 | null |
| Provatas | 15prm1 | 7,07E+05 | 3,22E+03 | 0,4558 | 1,8963 | null |
| Thermia | 49the-1 | 1,08E+06 | 1,27E+04 | 1,1759 | 4,8919 | null |
| Traianoupoli | 6tra1 | 5,62E+06 | 4,66E+04 | 0,8298 | 3,4521 | null |
| Traianoupoli | 7tra2 | 3,60E+06 | 4,52E+05 | 12,5544 | 52,2264 | null |
| Tria Pigadia | 27tpm2 | 5,20E+06 | 7,05E+02 | 0,0136 | 0,0564 | null |

## Supplementary table 4

| Taxonomy | 4NAP-1  (multiple) | col  (single) | 49THE-1  (single) | 51THE-3  (single) | 44AGP-2  (null) | 25KAM-6  (null) | NEFER  (null) | NESUL  (null) |
| --- | --- | --- | --- | --- | --- | --- | --- | --- |
| *Anaerostipes* | 76 | 5 | 16 | 2 | 4 | 2 | 21 | 6 |
| *Anoxybacillus* | 3 | 0 | 0 | 0 | 0 | 0 | 0 | 0 |
| *Bacillus* | 33 | 1 | 1237 | 2 | 494 | 340 | 391 | 37 |
| *Brevibacillus* | 27 | 0 | 0 | 0 | 0 | 0 | 0 | 0 |
| *Clostridium* | 3544 | 21 | 1493 | 1939 | 2165 | 38 | 1983 | 395 |
| *Desulfotomaculum* | 2 | 1 | 0 | 13 | 0 | 0 | 7 | 1 |
| *Lysinibacillus* | 25 | 0 | 0 | 2 | 0 | 0 | 2 | 7 |
| *Paenibacillus* | 27 | 0 | 0 | 0 | 0 | 0 | 0 | 0 |

## Supplementary Figure 1


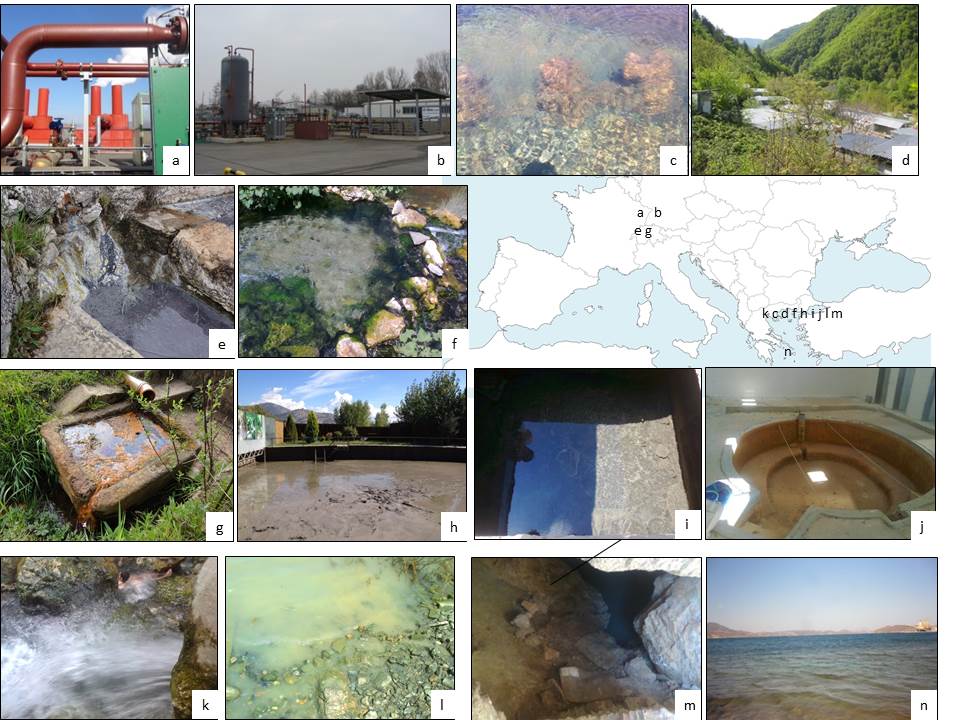


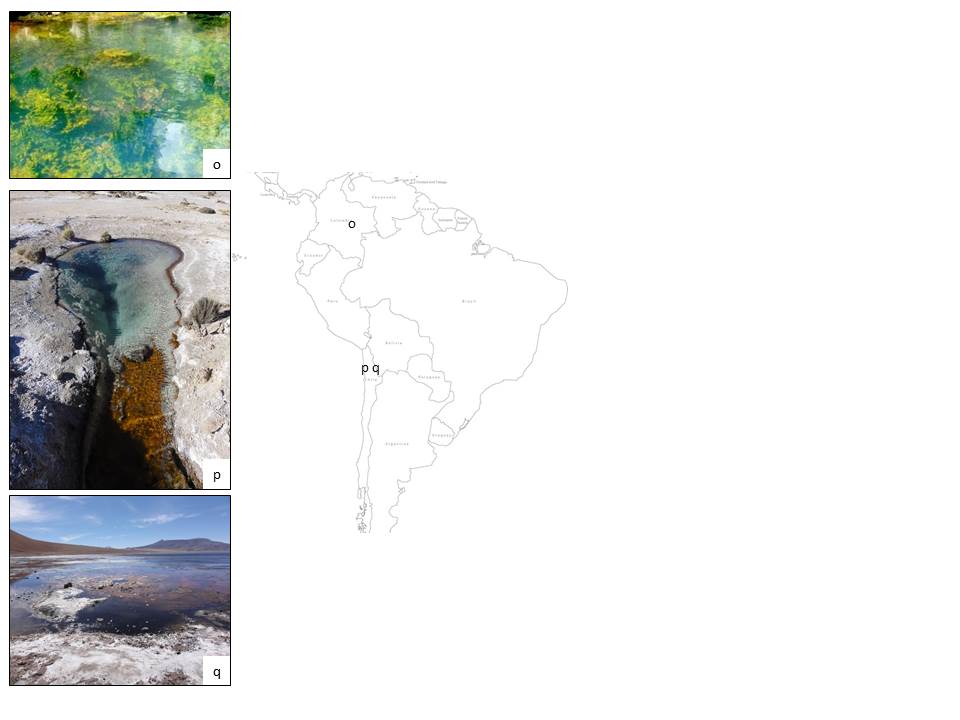


## Supplementary Figure 1 Sampling sites worldwide. (A) Map showing the countries in Europe from which samples were collected, as well as pictures of the geothermal springs and reservoirs. Clockwise from top left: France: Soultz-sous-forets; Germany: Bruschal; Greece: Nea Apollonia, Aggistro, Thermia, Eleftheres, Krinides, Nigrita, Milos, Agia Paraskevi, Pikrolimni, Pozar, Switzerland: Ponts-de-Martel: iron spring, sulfur spring; (B) Map showing the sampling points along with pictures from the sampling sites in Colombia (Los Volcanes) and Chile (Lirima (top), Aguas Calientes (bottom)).

## Supplementary Figure 2

**Supplementary Figure 2.** DNA isolated in ng per type of sample.

##

**Supplementary Figure 3.** Abundance of Firmicutes compared to the rest of the bacterial community.

## Script

#The following libraries were run upon initiation of the analysis

library(lattice)

library(car)

library(multcomp)

library(gplots)

library(effects)

library(MASS

library(nlme)

###############################

# Quantitative data

###############################

library(RODBC)

dat = read.table("fil_quanty.txt", sep="\t", header=TRUE)

head(dat)

tail(dat)

summary(dat)

dim(dat)

names(dat)

dat$ratio <- dat$ratio/100

dim(dat[dat$ratio<=1,])-dim(dat)

dat[dat$ratio > 1,] # the value bigger than 1

dat[15,]$ratio <- 1 # replace the value bigger than 1

dat$ratio.tr <- asin(sqrt(dat$ratio)) # transformed data

hist(dat$ratio.tr, col="mistyrose")

dat0 <- dat[,c(8:10,12)] # only variables of interest

# Visualize data

pairs(dat0, panel = panel.smooth,

cex = 1.5, pch = 24, bg = "light blue",

diag.panel = panel.hist, cex.labels = 2, font.labels = 2)

# Tree method

require(party)

plot(ctree(ratio.tr ~ ph+HTM+Temp, data=dat))

plot(ctree(DNA ~ ph+HTM+Temp, data=na.omit(dat[,c(4,8:10)])))

# Some mean/vars in each group

bwplot(ratio.tr ~ Location, data=dat)

bwplot(ratio.tr ~ Source, data=dat)

# Generalized additive model

require(mgcv)

plot(gam(ratio.tr ~ s(Temp), data=dat), residuals=T, pch=16, pages=1, shade=T)

plot(gam(ratio.tr ~ s(ph), data=dat), residuals=T, pch=16, pages=1, shade=T)

plot(gam(ratio.tr ~ s(HTM), data=dat), residuals=T, pch=16, pages=1, shade=T)

# Full correlation matrix

cor(dat0, method="spearman", use="pairwise.complete.obs")

# Corelation ratio-Temperature

# Scatterplot

x <- dat$Temp

y <- dat$ratio

sequence <- order(x)

plot(x, y, pch=16, col=2, ylab="ratio", xlab="Temp")

lines(x[sequence],y[sequence], col=3)

dim(na.omit(cbind.data.frame(dat$ratio.tr, dat$Temp)))[1] # number of pairwise complete obs

cor(dat$ratio.tr, dat$Temp, use="pairwise.complete.obs")^2 # R-squared

cor.test(dat$ratio.tr, dat$Temp)

cor.test(dat$ratio.tr, dat$Temp, method = "spearman", continuity=F)

cor.test(dat$ratio.tr, dat$Temp, method = "kendall", continuity=F)

cor.test(dat[dat$Temp >=12 & dat$Temp <=60 & dat$ratio <=0.004,]$ratio.tr,

dat[dat$Temp >=12 & dat$Temp <=60 & dat$ratio <=0.004,]$Temp,

method = "spearman", continuity=F) # correlation on the subset of "normal temperature"

# Correlation log(DNA)-Temperature

hist(log(dat$DNA+1), col="mistyrose")

cor.test(log(dat$DNA+1), dat$Temp, method = "spearman")

# Scatterplot

y <- log(dat$DNA+1)

x <- dat$Temp

sequence <- order(x)

plot(x, y, pch=16, col=2, ylab="DNA log transformed", xlab="Temp")

lines(x[sequence],y[sequence], col=3)

# Correlation ratio-HTM

# Scatterplot

x <- dat$HTM

y <- dat$ratio.tr

sequence <- order(x)

plot(x, y, pch=16, col=2, ylab="ratio", xlab="HTM")

lines(x[sequence],y[sequence], col=3)

dim(na.omit(cbind.data.frame(dat$ratio.tr, dat$HTM)))[1] # number of pairwise complete obs

cor(dat$ratio.tr, dat$HTM, use="pairwise.complete.obs")^2 # R-squared

cor.test(dat$ratio.tr, dat$HTM)

cor.test(dat$ratio.tr, dat$HTM, method = "spearman", continuity=F)

cor.test(dat$ratio.tr, dat$HTM, method = "kendall", continuity=F)

lm0 <- lm(ratio.tr ~ HTM, data=dat[-c(3,5),]) # 3,5 are outliers

summary(lm0)

# Check model assumptions

par(mfrow=c(2,2));plot(lm0); par(mfrow=c(1,1))

plot(dat$HTM, dat$ratio.tr, col="red"); abline(lm0, col="blue")

# Corelation ratio-pH

# Scatterplot

x <- dat$ph

y <- dat$ratio.tr

sequence <- order(x)

plot(x, y, pch=16, col=2, ylab="ratio", xlab="ph")

lines(x[sequence],y[sequence], col=3)

dim(na.omit(cbind.data.frame(dat$ratio.tr, dat$ph)))[1] # number of pairwise complete obs

cor(dat$ratio.tr, dat$ph, use = "pairwise.complete.obs")^2 # R-squared

cor.test(dat$ratio.tr, dat$ph)

cor.test(dat$ratio.tr, dat$ph, method = "spearman", continuity=F)

cor.test(dat$ratio.tr, dat$ph, method = "kendall", continuity=F)

###############################

# Qualitative data

##############################

library(RODBC)

#datfile = "fil_quali.xlsx" # get the data path

#getxlsbook = odbcConnectExcel2007(datfile) # link the excel data book

#dat2 = sqlFetch(getxlsbook, "Sheet2") # get the data from the data sheet

#odbcCloseAll()

dat2=read.table("fil_quali.txt", sep="\t", header=TRUE)

head(dat2)

summary(dat2)

dim(dat2)

names(dat2)

dat2 <- dat2[,-c(11,12,14)]

names(dat2)[7] <- "ratio"

dat2$ratio <- dat2$ratio/100

round(range(dat2$ratio),2)

dat2[118,]$ratio <- 1

hist(dat2$ratio, col="mistyrose")

dat2$ratio.tr <- asin(sqrt(dat2$ratio)) # transformed data

hist(dat2$ratio.tr, col="mistyrose")

# Transform Nofact in a factor (ordered)

dat2$Nofact <-as.factor(dat2$Nofact)

is.ordered(dat2$Nofact)

levels(dat2$Nofact)

dat2$Nofact <- as.ordered(dat2$Nofact) # test used to verify orthogonal polynomial contrasts

#levels(dat2$Nofact)[5] <- "4"

#levels(dat2$Nofact)[2] <- "2"

table(dat2$Nofact)

# Visualize data as Grouped Bar Plot

barplot(as.matrix(rbind(tapply(dat2$tot, dat2$Nofact, mean),tapply(dat2$mycro, dat2$Nofact, mean))),

main="Microorganism abundance",

xlab="Nb of extreme factors", col=c("darkblue","red"),

legend = c("total", "my organism"), beside=TRUE)

barplot(as.matrix(rbind(tapply(log(dat2$tot+1), dat2$Nofact, mean),tapply(log(dat2$mycro+1), dat2$Nofact, mean))),

main="Log-transformed Microorganism abundance",

xlab="Nb of extreme factors", col=c("darkblue","red"),

legend = c("total", "my organism"), beside=TRUE)

plot(levels(dat2$Nofact), tapply(dat2$tot, dat2$Nofact, mean), type="b", col = "darkblue")

plot(levels(dat2$Nofact), tapply(dat2$mycro, dat2$Nofact, mean), type="b", col= "red", add=TRUE)

plot(levels(dat2$Nofact), tapply(dat2$ratio, dat2$Nofact, mean), type="b", col= "orange", add=TRUE)

# Tree method

require(party)

plot(ctree(ratio.tr~ Nofact + HTM, na.omit(dat2[,c(10,12:13)])))

# Some mean/vars in each group

bwplot(log(tot+1) ~ Nofact, data=dat2[-c(84,89,90,103),]) # we excluded some extreme values from the plot

bwplot(ratio.tr~Nofact, data=dat2[-c(84,89,90,103),]) # we excluded some extreme values from the plot

bwplot(ratio.tr~Location, data=dat2)

bwplot(ratio.tr~Source, data=dat2)

# Classical ANOVA

model <- lm(ratio.tr ~ Nofact, data=dat2[dat2$Nofact!="8",])

summary(model)

# Check model assumptions

par(mfrow=c(2,2));plot(model); par(mfrow=c(1,1)) # not OK!! model is not appropriate

model1 <- gls(ratio.tr ~ Nofact, weights = varIdent(form=~1|Nofact), data=dat2[-c(40,52,53),])

summary(mmodel3)

# Check model assumptions

qqnorm(model1, abline=c(0,1), id = 0.05)

plot(model1)

plot(effect("Nofact", model1)) # you can use this plot even if the model is not quite OK

# Non-parametric tests of Nofact effect

require(agricolae)

comparison <- kruskal(dat2$ratio.tr, dat2$Nofact, group=TRUE, main="Relative abundance")

comparison

comparison1 <- kruskal(dat2$tot, dat2$Nofact, group=TRUE, main="Relative abundance")

comparison1

# Model for total abundace of microorganisms

mmodel3t <- gls(log(tot+1) ~ Nofact, weights = varIdent(form=~1|Nofact), data=dat2)

summary(mmodel3t)

# Check model assumptions

qqnorm(mmodel3t, abline=c(0,1), id = 0.05)

plot(mmodel3t)

plot(effect("Nofact", mmodel3t))

#a model that remove the variability between locations

mmodel <- lme(log(tot+1) ~ Nofact, random = ~ 1|Location, data=dat2)

summary(mmodel)

plot(mmodel)

mmodel1 <-update(mmodel, weights = varIdent(form=~1|Nofact) )

summary(mmodel1)

anova(mmodel,mmodel1) # improvement

# Check model assumptions

plot(mmodel1, resid(., type = "p") ~ fitted(.), id = 0.05)

qqnorm(mmodel1, abline=c(0,1), id = 0.05, col="blue")

qqnorm(mmodel1, ~ ranef(.))

plot(mmodel1, Location~resid(.), abline = 0 )

plot(mmodel1, resid(., type = "p") ~ fitted(.)|Location, id = 0.05)

require(lattice)

multiple.txt <- read.table("multiple2.txt", header=T)

head(multiple.txt)

single.txt <- read.table("single2.txt", header=T)

head(single.txt)

no.txt <- read.table("no2.txt", header=T)

head(no.txt)

summary(multiple.txt)

summary(single.txt)

summary(no.txt)

shapiro.test(multiple.txt$Multiple) # not normal

shapiro.test(single.txt$Single) # not normal

shapiro.test(no.txt$No_Factor) # not normal

varia <- c(multiple.txt$Multiple, single.txt$Single, no.txt$No_Factor)

treat <- c(rep("Multiple", length(multiple.txt$Multiple)),

rep("Single", length(single.txt$Single)),

rep("NoFact", length(no.txt$No_Factor)))

all <- cbind.data.frame(varia, treat)

summary(all)

boxplot(c(multiple.txt, single.txt, no.txt), ylab="qPCR ratio spo0A/16S", xlab = "sporulation triggering factors" )

bwplot(varia ~ treat, ylab="qPCR ratio spo0A/16S", xlab = "sporulation triggering factors")

(Means <- tapply(all$varia, all$treat, mean))

(Vars <- tapply(all$varia, all$treat, var))

plot(Means, Vars)

###############################################

# Non-parametric analysis

###############################################

# 1st version

require(agricolae)

cmp <- kruskal(all$varia, all$treat, group=T)

cmp

# 2nd version

require(PMCMR)

kruskal.test(all$varia~all$treat)

posthoc.kruskal.nemenyi.test(x=all$varia, g=all$treat, method="Tukey")
